# Host genetic diversity influences the severity of *Pseudomonas aeruginosa* pneumonia in the Collaborative Cross mice

**DOI:** 10.1186/s12863-015-0260-6

**Published:** 2015-08-28

**Authors:** Nicola Ivan Lorè, Fuad A Iraqi, Alessandra Bragonzi

**Affiliations:** Infection and Cystic Fibrosis Unit, Division of Immunology, Transplantation and Infectious Diseases, IRCCS - San Raffaele Scientific Institute, Milan, Italy; Department of Clinical Microbiology and Immunology, Sackler Faculty of Medicine, Tel Aviv University, Ramat Aviv, 69978 Tel Aviv Israel

**Keywords:** *P. aeruginosa*, Acute infection, CC mice, Disease phenotype, Airway infection, Animal model, Forward genetics

## Abstract

**Background:**

*Pseudomonas aeruginosa* is one of the top three causes of opportunistic infections in humans. Patients with a compromised immune system, due to immunosuppressive therapies or underlying diseases such as cancer, AIDS or the hereditary disease cystic fibrosis, are at risk of developing *P. aeruginosa* infection. However, clinical evidence indicates extremely variable outcomes of *P. aeruginosa* infections in individuals at risk, suggesting that host multi-complex genetic traits may influence the severity of this opportunistic infection. Here, we have used an innovative experimental model to dissect whether host genetic background, such as those found in the outbred population, could influence the risk of morbidity and mortality to *P. aeruginosa* pneumonia.

**Results:**

A highly genetically-diverse mouse resource population, Collaborative Cross (CC) mice, was infected with a clinical strain of *P. aeruginosa* and subsequently monitored for mortality, mean survival time, and morbidity, change in body weight for seven days post infection. Disease phenotypes ranged from complete resistance and recovery of body weight to lethal disease. Initial variables, including body weight, age and gender, have limited influence on *P. aeruginosa* outcome, emphasizing the role of host genetic background in defining the risk of morbidity and mortality. When broad-sense heritability of phenotypic traits was evaluated, it confirmed the influence of genetic profile rather than environmental factors among the CC lines during *P. aeruginosa* infection.

**Conclusion:**

This innovative model system can potentially reproduce the variables responses of disease severity observed in humans during *P. aeruginosa* pneumonia. Our results demonstrated that a widely-marked differential response to *P. aeruginosa* airway infection in term of morbidity and mortality, is mainly affected by host genetic factors, as multiple genetic *loci* or polymorphic variations.

**Electronic supplementary material:**

The online version of this article (doi:10.1186/s12863-015-0260-6) contains supplementary material, which is available to authorized users.

## Background

Genotype is an important determinant factor in human host susceptibility to major diseases, including infections [1]. There is increasing evidence that some inter-population and inter-individual differences in the attack rate and prognosis of specific infectious organisms are due to inherited genetic variants and, for the most part, to multicomplex genetic traits (polygenetic traits) [2]. These findings are changing our view of infections as we can now assume that pathogens are not the sole determinants of the corresponding infectious diseases. Host response is influenced by the complex combinations and variations of genes, and this affects the outcome of the infectious disease. An efficient experimental method to dissect complex genetic traits still needs to be established. Recently, a new community resource - Collaborative Cross (CC) mice - has been implemented as a common platform for mammalian complex genetic traits in an attempt to overcome the limitation of existing resources [3, 4]. The CC is a murine reference population with high genetic diversity, constructed using a randomized breeding design that systematically outcrosses eight founder strains, followed by inbreeding to obtain new recombinant inbred strains. Five of the eight founders are common laboratory strains (A/J, C57BL/6 J, 129S1/SvImJ, NOD/LtJ, NZO/HiLtJ), and three are wild-derived strains (CAST/EiJ, PWK/PhJ, and WSB/EiJ) [5]. The CC has been genotyped and showed more recombination and genetic variation compared to the other reference panels [6]. The genomes of the CC founder strains (http://www.sanger.ac.uk/resources/mouse/genomes/) and CC lines strains (http://csbio.unc.edu/CCstatus/index.py) (http://mus.well.ox.ac.uk/CC/) have been genotyped or sequenced, so it is possible to reconstruct the haplotypes of each CC line as a fixed mosaic of the founder chromosomes [6, 7]. Hallmarks of CC lines include high mapping resolution and sample sizes that are sufficient to drive phenotypic diversity in almost any trait of interest.

This paper reports the use of CC mice as an innovative forward genetic approach to determine whether and to what extent host genetic background, influences the variability of *P. aeruginosa* acute respiratory infection. *P. aeruginosa* retains a prominent position as a major worldwide cause of morbidity and mortality in a wide range of patients including those with a compromised immune system from immunosuppressive therapies or underlying diseases such as cancer, AIDS or the hereditary disease, cystic fibrosis (CF) [8–10]. Clinical evidence indicates that the outcome of *P. aeruginosa* infections may be extremely variable among individuals at risk, suggesting that host multicomplex genetic traits may influence the severity of this opportunistic infection. Genetic mapping in the human population has been performed, but partially achieved significance in the genome-wide association study [11]. Studies from CF twins and sibs have suggested that susceptibility to chronic infection with *P. aeruginosa* is affected by genetic inheritance determinants, whereas initial infection is minimally influenced by genetic modifiers [12]. However, the size of the cohorts, the strong but often unknown environmental influences, poor diagnosis and lack of repeatability are bottlenecks in human studies that highlight the need for novel resources [13–15]. Mouse inbred strains are the starting point from which to explore causal genotype-phenotype relationships and identify gene mapping however they do not offer a strong tool to identify and study polymorphic variations, that are hallmarks of the human population [16, 17]. The number of classical inbred strains is relatively small. This limits genetic and phenotypic diversity and the value of Quantitative Traits Loci (QTL) detection. Thus, innovative experimental animal models are absolutely essential to complement the human studies [18]. Using CC mice, it is possible to determine genotype variation associated to disease phenotypic traits (e.g., using merge analysis) [6, 7]. Power and resolution mapping of CC mice was recently completed for host susceptibility to *Aspergillus fumigatu*s [7], influenza A [19, 20], *Klebsiella* pneumoniae [21] and reported for the first time in this paper as proof-of-concept for new studies to map QTL associated with the opportunistic bacteria *P. aeruginosa*.

## Results

### Host genetic background influences the severity of *P. aeruginosa* pneumonia

**Fig 1:**
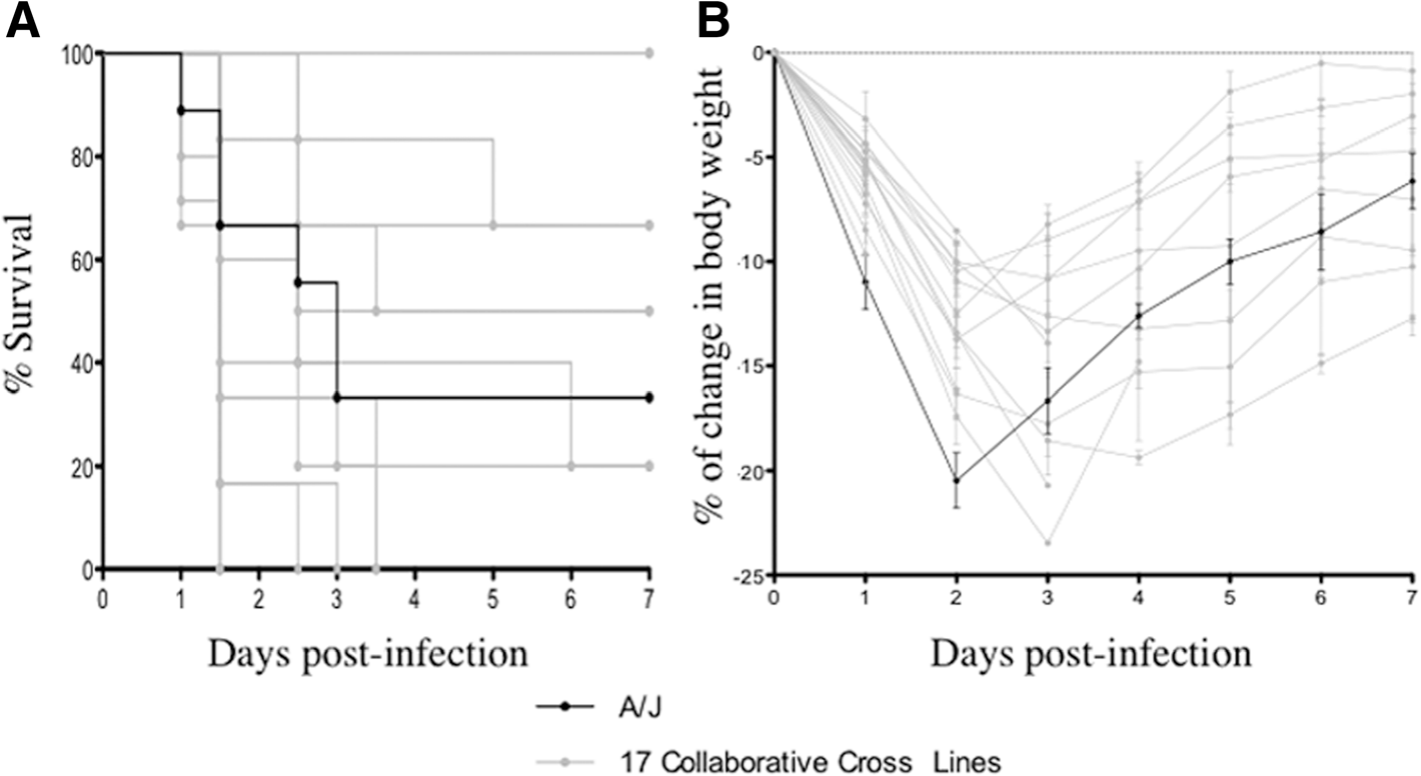
Disease phenotypes in CC mice during *P. aeruginosa* acute respiratory infection. 17 CC lines and A/J mice of 8–14 weeks old (between 3–7 mice per Line), were inoculated with a 1×10^6^ cfu dose of the *P. aeruginosa* clinical isolate AA2, and monitored for survival time ST (**a**) and body weight BW (**b**) for a period of 7 days after infection

CC lines, infected with *P. aeruginosa,* showed a wide-range of Survival time (ST) ranging from complete resistance (100 % survival after seven days post-infection) to lethal disease (100 % death after 1.5 days), while A/J mice showed an intermediate phenotype (30 % of mortality rate after 7 days post-infection) (Fig. 1a, Additional files 1 and 2). Similarly, CC lines had a wide variation in body weight (BW) response to *P. aeruginosa* infection: ranging from a 23 % decrease in BW after three days to those showing an almost total recovery of change in BW after five days (Fig. 1b). A/J mice lost 16 % of their change in BW after three days but they did not recover completely after seven days. At day 7 post-infection, bacterial cells were not recovered in the organs (blood, liver and lung) of surviving mice (data not shown), indicating that bacterial clearance is independent from differences in morbidity as assessed by recovery of body weight. These data confirm that different traits of the CC mice resource population express a wide response to *P. aeruginosa* infection, suggesting the key role of genetic variance on the severity of the clinical outcome.

### Segregation analysis of disease phenotypic traits

**Fig 2:**
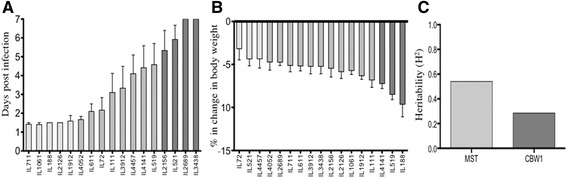
Evaluation of MST, CBW1 and H^2^ of CC lines after *P. aeruginosa* airway infection. The CC mice resource population had a strong wide-response to *P. aeruginosa* airway infection in the MST (**a**) and CBW1 traits (**b**). MST and CBW1 of CC lines are arranged in increasing order of mean magnitude. Based on Bonferroni's Multiple Comparison Tests (BMCT) (Additional file 3: Table S3) three distinct groups have been identified among 17 CC strains infected with *P. aeruginosa* and are indicated as scales of grey. Estimates of broad sense H^2^ (**c**) have been evaluated for MST and CBW1, as previously described [23]

Phenotypic traits for segregation analysis of mean survival time (MST) and change in body weight at day one (CBW1) after *P. aeruginosa* airway infection were selected based on previous studies with commercial inbred strains [17, 22]. Thus, segregation analysis of the phenotypic trait distributions highlights the different responses among CC lines. Figure 2 shows MST and CBW1 of the CC lines, arranged by increasing order of magnitude. Based on the statistical significance using Bonferroni’s Multiple Comparison Tests (reported in Additional file 3), CC lines were arranged by a similar variation degree of recorded traits in three groups (Fig. 2). Thus, in the high tail of MST, four CC lines (IL2156, IL521, IL2689, IL3438) were ranked with the highest MST value (MST: 5.3/7) while in the low tail, five CC lines (IL711, IL1061, IL188, IL2126, IL1912) showed the lowest MST values (MST: 1.41/1.48) (Fig. 2a). An intermediate group of eight murine lines (IL4052, IL611, IL72, IL111, IL3912, IL4457, IL4141, IL519) showed a medium MST value (MST: 1.6/4.5). Statistical analysis of CBW1 highlights a two-fold difference between the highest (IL4141, IL519, IL188; CBW1: −7.22 %/−9.33 %) and the lowest CC line groups (IL72, IL521, IL4457; CBW1: −3.16 %/−4.34 %) (Fig. 2b). An intermediate group of eleven murine lines (IL4052, IL2689, IL711, IL611, IL3912, IL3438, IL2156, IL2126, IL1061, IL1912, IL111; CBW1: −4.70 %/−6.80 %) was also recorded. Overall our data show a wide range of responses to *P. aeruginosa* infection, potentially caused by allelic segregations from the eight founders strains.

### Effect of initial body weight, age and gender on disease phenotypes

The effects of initial body weight or age on ST, CBW1 were tested across 17 CC lines and A/J mice; they were found not to be significant by Spearman‘s Correlation (Table 1A). Similarly, gender effect on ST and CBW1 was not significantly correlated when the whole murine population was considered. Within individual CC lines no significant gender effect on disease phenotypes was observed among male and female mice (Table 1B), although a limited number of animals was consider. All together these results, emphasize the key role of genetic determinants in the severity and in the outcome of *P. aeruginosa* airway infections using this diverse murine population.

**Table 1:** Analysis of initial body weight, age and gender on disease phenotypes

A	Survival Time (n = 92)			Change in BW day 1 (n = 92)			
Initial body weight	*r* = 0.192 P > 0.05			*r* = 0.010 P > 0.05			
Week’s old	*r* = 0.099 P > 0.05			*r* = 0.173 P > 0.05			
Gender	*r* = 0.070 P > 0.05			*r* = 0.035 P > 0.05			
B							
Mean Survival Time							
	Male			Female			ANOVA
	Mean	SEM	n	Mean	SEM	n	P value
A/J	1.83	0.60	3	4.67	1.06	6	P > 0.05
IL 4052	1.83	0.33	3	1.50	0.00	3	P > 0.05
IL 3438	7.00	0.00	3	7.00	0.00	3	P > 0.05
IL 2156	7.00	0.00	3	3.67	1.69	3	P > 0.05
IL 711	1.50	0.00	2	1.30	0.12	5	P > 0.05
IL 2126	1.50	0.00	4	1.50	0.00	2	P > 0.05
IL 4141	4.25	2.75	2	4.50	1.46	4	P > 0.05
IL 4457	5.17	1.36	3	2.50	0.00	2	P > 0.05
IL 1912	1.17	0.17	3	2.00	0.50	3	P > 0.05
% Change in body weight day 1							
	Male			Female			ANOVA
	Mean	SEM	n	Mean	SEM	n	P value
A/J	−10.53	5.90	2	−11.30	1.11	5	P > 0.05
IL 4052	−3.05	0.94	3	−6.37	0.91	3	P > 0.05
IL 3438	−6.52	0.79	3	−4.28	0.47	3	P > 0.05
IL 2156	−5.69	1.75	3	−5.18	1.41	3	P > 0.05
IL 711	−5.13	1.30	2	−5.10	1.23	2	P > 0.05
IL 2126	−6.18	1.19	4	−5.15	0.49	2	P > 0.05
IL 4141	−8.45	1.27	2	−6.62	0.43	4	P > 0.05
IL 4457	−3.76	1.41	3	−5.23	2.03	2	P > 0.05
IL 1912	−6.36	0.00	1	−6.29	0.59	3	P > 0.05

Overall Spearman‘s correlation (*r* correlation coefficients and *P* value) between all the initial parameters (initial BW, week’s old and gender) of CC lines and recorded traits (ST: n = 92, CBW1: n = 92) to determine potential influence (**A**). Two-way ANOVA’s comparison across eight selected CC lines and A/J commercial inbred line to evaluate gender influence on recorded traits (MST and CWB1) (**B**)

### Heritability of disease phenotypes during *P. aeruginosa* pneumonia

To test if the traits variations within the infected CC murine population were due to genetic factors, we estimated heritability as described [23, 24]. Broad-sense heritability (H^2^) was calculated for all the CC lines for which MST and CBW1 were recorded (Fig. 2c). Estimated H^2^ was 0.54 and 0.28 for MST and CBW1, respectively. Thus, on observation of the CC lines during *P. aeruginosa* infection, there is a clear host response that is affected by the genetic components as opposed to the purely environmental factors.

## Discussion

Several studies highlight how host response to infection may be strongly influenced by the cumulative effect and interactions of multiple genetic loci and by a complex set of other factors (e.g. the environment, the bacterial strain and origin, age and gender) [1, 13, 25]. Among the most relevant infectious diseases that affect humans, *P. aeruginosa* shows a wide variation in the clinical outcome in individuals at risk [8–10], indicating that the host-response may contribute to the variation of morbidity and mortality. However, predictive experimental animal models to dissect complex genetic traits, such as multiple genetic loci, that can influence the outcome of *P. aeruginosa* respiratory infection, remain to be established. To date, a poor phenotype/genotype correlation in human studies and the lack of a fully faithful mouse model have limited scientific advancements in the field [13–15]. To meet the current challenge of complex trait analysis, we used CC mice to evaluate the contribution of the host to *P. aeruginosa* pathogenicity. First, based on phenotypic diversity of human response to infection, we defined two end-points (ST and BW) that may ultimately have clinical relevance in term of morbidity and mortality. Based on previous studies with commercial inbred strains [17, 22], MST and CBW1 were selected as phenotypic disease traits to perform segregation analysis. Our approach generated wide-marked range of phenotypic differences in terms of MST and CBW1 during *P. aeruginosa* pneumonia, although a limited number of lines were considered. On the other, future experiments will define detailed phenotypic traits, such as bacterial load, inflammatory cytokines or cellular recruitment into the lung.

Next, initial variables - including body weight, age and gender - have limited influence on *P. aeruginosa* outcome, emphasizing the key role of genetic determinants in the disease severity in our infectious model system. In addition, the high value of the broad-sense heritability of the two recorded disease phenotypic traits support our suggestion that host genotype is an important determinant factor in the disease’s severity. A similar approach was developed using Diversity Outbred mice to model *Staphylococcus aureus* and influenza Virus pneumonia [26]. Interestingly, they demonstrated that initial body weight correlated with viral burden, including co-infected mice, but not with *S. aureus* bacterial load. These suggest that the correlation of initial body weight and infectious graveness is modulated by the specific host-pathogen interplay. Both host genetic background and type of pathogen may have a key role in determining morbidity.

Of relevance, the power of CC population as model system relies also in the fact that all potential co-variables, that may influence phenotypic traits, are taken into account in the statistical analysis. Thus for future mapping of key genetic loci/genes, by using CC population and model of airway infection, we can track the influence of confounding effects that are bottlenecks in genetic mapping in the human population.

## Conclusions

Exploring CC mouse population together with a model of respiratory infection, our results demonstrates that *P. aeruginosa* opportunistic infection has wide range of disease phenotypes affected by multiple host genetic factors, such as multiple genetic loci or polymorphic variations. Future mapping of key genetic loci/genes involved in the *P. aeruginosa* infection will be carried out with the use of additional CC lines and further traits of disease phenotypes will be assessed. This innovative approach, based on the concept of forward genetics, will provide new insight into the key molecular processes that control host/pathogen interactions in respiratory disease, and should reveal novel targets for human personalized therapeutic strategies.

## Material and methods

### Ethic statement

All experimental mice and protocols were approved by the Institutional Animal Care and Use Committee of Tel Aviv University (TAU) (approval number: M-13-079). *P. aeruginosa* clinical isolate AA2 was obtained from CF patient attending the Medizinische Hochschule of Hannover, Germany and described previously [17, 22, 27]. Research on the bacterial isolates from the individual with CF has been approved by the responsible physician at the CF center at Hannover Medical School, Germany. Patient gave informed consent before the sample collection. Approval for storing of biological materials was obtained by the Hannover Medical School, Germany.

### Collaborative cross and inbred lines

A total of 92 (50 Males, 42 Females) mice (8 to 14-week old) from 17 different CC mouse lines (average 3–7 mice per line) were provided by the Small Animal Facility at Sackler Faculty of Medicine, Tel Aviv University (TAU). The lines were at inbreeding generations F20-F39, minimum 90 % homozygosity. Full details of the development of these CC lines are given in Welsh et al. [3, 28]. In addition to the CC mice, commercially inbred lines, A/J mice were used as internal control and were was purchased from Jackson Laboratory. The infection challenge was carried out at the BSL-2 laboratory at TAU. Mice were housed on hardwood chip bedding in open-top cages at the animal facility and were given tap water and rodent chow ad libitum.

After *P. aeruginosa* infection, mice were monitored twice per day for the parameters vocalisation, piloerection, attitude, locomotion, breathing, curiosity, nasal secretion, grooming and dehydration. Mice that lost >25 % body weight and had evidence of severe clinical disease, such as scruffy coat, inactivity, loss of appetite, poor locomotion, or painful posture, were sacrificed before the termination of the experiments with an overdose of carbon dioxide.

### *P. aeruginosa* strain and mouse model of acute respiratory infection

Prior to the animal experiments, *P. aeruginosa* AA2 clinical strain was grown in Trypticase Soy Broth (TSB) to reach the exponential phase at 37 °C [22, 27]. The mice were anesthetized and infected by intratracheal injection with a 10^6^ colony forming unit (cfu) implanted into the lung via the cannula, with all lobes inoculated as described [22]. ST and BW of mice were monitored daily over one week; then the surviving mice were euthanized and tested for cfu in blood, liver and lung.

### Disease phenotypic traits and segregation analysis

ST and BW of mice were considered as disease phenotypic traits for mortality and morbidity of *P. aeruginosa* pneumonia. In particular, MST and CBW1 were selected for segregation analysis, based on previous studies with commercial inbred strains [17, 22]. At day one post *P. aeruginosa* infection, a high decrease of body weight associated to low mortality rate was reported [17, 22]. Based on the statistical significance, using Bonferroni’s Multiple Comparison Tests (reported in Additional file 3), CC lines were arranged in three groups for MTS and CBW1 by increasing order of magnitude, as described [24].

### Estimation of heritability

Broad sense heritability - including epistatic but not dominance effects for MST and CBW1 - was calculated across the CC lines under *P. aeruginosa* infection and in naïve conditions as described [23]. Briefly, One-way ANOVA by CC line was implemented separately for MST and CBW1. Based on these analyses, broad sense heritability (H^2^), was calculated across the CC lines under control and challenge conditions, separately.

### Statistical analysis

Spearman‘s correlation was used for associations tests. One- and Two-ANOVA and Bonferroni’s Multiple Comparison Tests were used to determine the statistical significance, using GraphPad software.

## Additional files

Additional file 1: Table S1. Survival Time of each CC lines after *P. aeruginosa* airway infection. (DOCX 51 kb) Additional file 2: Table S2. Change in body weight of each CC line after *P. aeruginosa* airway. (DOCX 51 kb) Additional file 3: Table S3. Bonferroni’s Multiple Comparison Tests of recorded traits (MST and CBW1) among CC lines after *P. aeruginosa* airway infection. (DOCX 74 kb)

## Abbreviations

CC: Collaborative Cross
ST: Survival time
MST: Mean survival time
BW: Body weight
CBW1: Change in body weight at day 1
QTL: Quantitative trait loci
