# Supplementary material for: Host genetic diversity influences the severity of Pseudomonas aeruginosa pneumonia in the Collaborative Cross mice
Source: BMC Genet. 2015 Aug 28;16:106. doi: 10.1186/s12863-015-0260-6 (PMC4551369; doi:10.1186/s12863-015-0260-6)
Supplement: Additional file 1: Table S1. — Survival Time of each CC lines after P. aeruginosa airway infection. (DOCX 51 kb) [file 12863_2015_260_MOESM1_ESM.docx]

**Host genetic diversity underlines the severity of *Pseudomonas aeruginosa* pneumonia in the Collaborative Cross mice**

Lore’ NI^1^, Iraqi FA^2^, Bragonzi A^1^

^1^ Infection and Cystic Fibrosis Unit, IRCCS - San Raffaele Scientific Institute, Milano, Italy

^2^ Department of Clinical Microbiology and Immunology, Sackler Faculty of Medicine, Tel Aviv University, Ramat Aviv, 69978, Tel Aviv, Israel,

Online Data Supplement

**Table S1:** *Survival Time of each CC line after P. aeruginosa airway infection.*

| **CC.Line** | **n. of Mice** | **Mean ST** | **Std. Deviation** | **Std. Error** |
| --- | --- | --- | --- | --- |
| IL711 | 7 | 1,357 | 0,244 | 0,09221 |
| IL1061 | 5 | 1,4 | 0,2236 | 0,1 |
| IL188 | 3 | 1,5 | 0 | 0 |
| IL2126 | 6 | 1,5 | 0 | 0 |
| IL1912 | 6 | 1,583 | 0,736 | 0,3005 |
| IL4052 | 6 | 1,667 | 0,4082 | 0,1667 |
| IL611 | 5 | 2,1 | 0,8944 | 0,4 |
| IL72 | 3 | 2,167 | 1,155 | 0,6667 |
| IL111 | 5 | 3,1 | 2,275 | 1,017 |
| IL3912 | 6 | 3,333 | 2,84 | 1,16 |
| IL4457 | 5 | 4,1 | 2,219 | 0,9925 |
| IL4141 | 6 | 4,417 | 2,853 | 1,165 |
| IL519 | 6 | 4,583 | 2,746 | 1,121 |
| IL2156 | 6 | 5,333 | 2,601 | 1,062 |
| IL521 | 6 | 5,917 | 1,855 | 0,7574 |
| IL3438 | 6 | 7 | 0 | 0 |
| IL2689 | 5 | 7 | 0 | 0 |
