# Supplementary material for: Host genetic diversity influences the severity of Pseudomonas aeruginosa pneumonia in the Collaborative Cross mice
Source: BMC Genet. 2015 Aug 28;16:106. doi: 10.1186/s12863-015-0260-6 (PMC4551369; doi:10.1186/s12863-015-0260-6)
Supplement: Additional file 2: Table S2. — Change in body weight of each CC line after P. aeruginosa airway. (DOCX 51 kb) [file 12863_2015_260_MOESM2_ESM.docx]

**Host genetic diversity underlines the severity of *Pseudomonas aeruginosa* pneumonia in the Collaborative Cross mice**

Lore’ NI^1^, Iraqi FA^2^, Bragonzi A^1^

^1^ Infection and Cystic Fibrosis Unit, IRCCS - San Raffaele Scientific Institute, Milano, Italy

^2^ Department of Clinical Microbiology and Immunology, Sackler Faculty of Medicine, Tel Aviv University, Ramat Aviv, 69978, Tel Aviv, Israel,

Online Data Supplemen

**Table S2:** *Change in body weight of each CC line after P. aeruginosa airway infection .*

| **CC.Line** | **n. of Mice** | **Mean CBW1** | **Std. Deviation** | **Std. Error** |
| --- | --- | --- | --- | --- |
| IL519 | 6 | -8,489 | 1,456 | 0,5944 |
| IL521 | 6 | -4,354 | 1,971 | 0,8048 |
| IL111 | 5 | -6,801 | 1,922 | 0,8595 |
| IL611 | 5 | -5,172 | 1,27 | 0,568 |
| IL711 | 4 | -5,114 | 1,464 | 0,732 |
| IL2126 | 6 | -5,834 | 1,944 | 0,7935 |
| IL2156 | 6 | -5,438 | 2,482 | 1,013 |
| IL2689 | 5 | -4,73 | 0,8754 | 0,3915 |
| IL3438 | 6 | -5,211 | 2,159 | 0,8814 |
| IL3912 | 6 | -5,211 | 2,159 | 0,8814 |
| IL4052 | 6 | -4,709 | 2,315 | 0,9452 |
| IL4141 | 6 | -7,225 | 1,406 | 0,5742 |
| IL4457 | 5 | -4,347 | 2,388 | 1,068 |
| IL72 | 3 | -3,167 | 2,246 | 1,297 |
| IL188 | 3 | -9,634 | 2,52 | 1,455 |
| IL1912 | 4 | -6,306 | 0,8358 | 0,4179 |
| IL1061 | 4 | -5,707 | 0,8903 | 0,4451 |
