# Supplementary material for: Host genetic diversity influences the severity of Pseudomonas aeruginosa pneumonia in the Collaborative Cross mice
Source: BMC Genet. 2015 Aug 28;16:106. doi: 10.1186/s12863-015-0260-6 (PMC4551369; doi:10.1186/s12863-015-0260-6)
Supplement: Additional file 3: Table S3. — Bonferroni’s Multiple Comparison Tests of recorded traits (MST and CBW1) among CC lines after P. aeruginosa airway infection. (DOCX 74 kb) [file 12863_2015_260_MOESM3_ESM.docx]

**Host genetic diversity underlines the severity of *Pseudomonas aeruginosa* pneumonia in the Collaborative Cross mice**

Lore’ NI^1^, Iraqi FA^2^, Bragonzi A^1^

^1^ Infection and Cystic Fibrosis Unit, IRCCS - San Raffaele Scientific Institute, Milano, Italy

^2^ Department of Clinical Microbiology and Immunology, Sackler Faculty of Medicine, Tel Aviv University, Ramat Aviv, 69978, Tel Aviv, Israel,

Online Data Supplemen

**Table S3:** *Bonferroni's Multiple Comparison Tests of recorded traits (MST and CBW1) among CC lines after P. aeruginosa airway infection* (ns P>0.05; *P≤0.05; **P ≤ 0.01; ***P ≤ 0.001; ****P ≤ 0.0001).

| **CC lines** | **Mean survival time** | | |
| --- | --- | --- | --- |
|  | **Mean Diff.** | **t** | **Significance** |
| IL711 vs IL1061 | 0.01667 | 0.01596 | ns |
| IL711 vs IL188 | -0.08333 | 0.06835 | ns |
| IL711 vs IL2126 | -0.08333 | 0.08371 | ns |
| IL711 vs IL1912 | -0.1667 | 0.1674 | ns |
| IL711 vs IL4052 | -0.25 | 0.2511 | ns |
| IL711 vs IL611 | -0.6833 | 0.6545 | ns |
| IL711 vs IL72 | -0.75 | 0.6151 | ns |
| IL711 vs IL111 | -1.683 | 1.612 | ns |
| IL711 vs IL3912 | -1.917 | 1.925 | ns |
| IL711 vs IL4457 | -2.683 | 2.57 | ns |
| IL711 vs IL4141 | -3 | 3.014 | ns |
| IL711 vs IL519 | -3.167 | 3.181 | ns |
| IL711 vs IL2156 | -3.917 | 3.934 | * |
| IL711 vs IL521 | -4.5 | 4.52 | ** |
| IL711 vs IL3438 | -5.583 | 5.609 | **** |
| IL711 vs IL2689 | -5.583 | 5.348 | *** |
| IL1061 vs IL188 | -0.1 | 0.07941 | ns |
| IL1061 vs IL2126 | -0.1 | 0.09578 | ns |
| IL1061 vs IL1912 | -0.1833 | 0.1756 | ns |
| IL1061 vs IL4052 | -0.2667 | 0.2554 | ns |
| IL1061 vs IL611 | -0.7 | 0.6419 | ns |
| IL1061 vs IL72 | -0.7667 | 0.6088 | ns |
| IL1061 vs IL111 | -1.7 | 1.559 | ns |
| IL1061 vs IL3912 | -1.933 | 1.852 | ns |
| IL1061 vs IL4457 | -2.7 | 2.476 | ns |
| IL1061 vs IL4141 | -3.017 | 2.889 | ns |
| IL1061 vs IL519 | -3.183 | 3.049 | ns |
| IL1061 vs IL2156 | -3.933 | 3.767 | * |
| IL1061 vs IL521 | -4.517 | 4.326 | ** |
| IL1061 vs IL3438 | -5.6 | 5.364 | *** |
| IL1061 vs IL2689 | -5.6 | 5.135 | *** |
| IL188 vs IL2126 | 0 | 0 | ns |
| IL188 vs IL1912 | -0.08333 | 0.06835 | ns |
| IL188 vs IL4052 | -0.1667 | 0.1367 | ns |
| IL188 vs IL611 | -0.6 | 0.4765 | ns |
| IL188 vs IL72 | -0.6667 | 0.4735 | ns |
| IL188 vs IL111 | -1.6 | 1.271 | ns |
| IL188 vs IL3912 | -1.833 | 1.504 | ns |
| IL188 vs IL4457 | -2.6 | 2.065 | ns |
| IL188 vs IL4141 | -2.917 | 2.392 | ns |
| IL188 vs IL519 | -3.083 | 2.529 | ns |
| IL188 vs IL2156 | -3.833 | 3.144 | ns |
| IL188 vs IL521 | -4.417 | 3.622 | ns |
| IL188 vs IL3438 | -5.5 | 4.511 | ** |
| IL188 vs IL2689 | -5.5 | 4.368 | ** |
| IL2126 vs IL1912 | -0.08333 | 0.08371 | ns |
| IL2126 vs IL4052 | -0.1667 | 0.1674 | ns |
| IL2126 vs IL611 | -0.6 | 0.5747 | ns |
| IL2126 vs IL72 | -0.6667 | 0.5468 | ns |
| IL2126 vs IL111 | -1.6 | 1.532 | ns |
| IL2126 vs IL3912 | -1.833 | 1.842 | ns |
| IL2126 vs IL4457 | -2.6 | 2.49 | ns |
| IL2126 vs IL4141 | -2.917 | 2.93 | ns |
| IL2126 vs IL519 | -3.083 | 3.097 | ns |
| IL2126 vs IL2156 | -3.833 | 3.851 | * |
| IL2126 vs IL521 | -4.417 | 4.437 | ** |
| IL2126 vs IL3438 | -5.5 | 5.525 | **** |
| IL2126 vs IL2689 | -5.5 | 5.268 | *** |
| IL1912 vs IL4052 | -0.08333 | 0.08371 | ns |
| IL1912 vs IL611 | -0.5167 | 0.4948 | ns |
| IL1912 vs IL72 | -0.5833 | 0.4784 | ns |
| IL1912 vs IL111 | -1.517 | 1.453 | ns |
| IL1912 vs IL3912 | -1.75 | 1.758 | ns |
| IL1912 vs IL4457 | -2.517 | 2.41 | ns |
| IL1912 vs IL4141 | -2.833 | 2.846 | ns |
| IL1912 vs IL519 | -3 | 3.014 | ns |
| IL1912 vs IL2156 | -3.75 | 3.767 | * |
| IL1912 vs IL521 | -4.333 | 4.353 | ** |
| IL1912 vs IL3438 | -5.417 | 5.441 | **** |
| IL1912 vs IL2689 | -5.417 | 5.188 | *** |
| IL4052 vs IL611 | -0.4333 | 0.415 | ns |
| IL4052 vs IL72 | -0.5 | 0.4101 | ns |
| IL4052 vs IL111 | -1.433 | 1.373 | ns |
| IL4052 vs IL3912 | -1.667 | 1.674 | ns |
| IL4052 vs IL4457 | -2.433 | 2.331 | ns |
| IL4052 vs IL4141 | -2.75 | 2.762 | ns |
| IL4052 vs IL519 | -2.917 | 2.93 | ns |
| IL4052 vs IL2156 | -3.667 | 3.683 | ns |
| IL4052 vs IL521 | -4.25 | 4.269 | ** |
| IL4052 vs IL3438 | -5.333 | 5.357 | *** |
| IL4052 vs IL2689 | -5.333 | 5.108 | *** |
| IL611 vs IL72 | -0.06667 | 0.05294 | ns |
| IL611 vs IL111 | -1 | 0.917 | ns |
| IL611 vs IL3912 | -1.233 | 1.181 | ns |
| IL611 vs IL4457 | -2 | 1.834 | ns |
| IL611 vs IL4141 | -2.317 | 2.219 | ns |
| IL611 vs IL519 | -2.483 | 2.378 | ns |
| IL611 vs IL2156 | -3.233 | 3.097 | ns |
| IL611 vs IL521 | -3.817 | 3.655 | ns |
| IL611 vs IL3438 | -4.9 | 4.693 | ** |
| IL611 vs IL2689 | -4.9 | 4.493 | ** |
| IL72 vs IL111 | -0.9333 | 0.7412 | ns |
| IL72 vs IL3912 | -1.167 | 0.9569 | ns |
| IL72 vs IL4457 | -1.933 | 1.535 | ns |
| IL72 vs IL4141 | -2.25 | 1.845 | ns |
| IL72 vs IL519 | -2.417 | 1.982 | ns |
| IL72 vs IL2156 | -3.167 | 2.597 | ns |
| IL72 vs IL521 | -3.75 | 3.076 | ns |
| IL72 vs IL3438 | -4.833 | 3.964 | * |
| IL72 vs IL2689 | -4.833 | 3.838 | * |
| IL111 vs IL3912 | -0.2333 | 0.2235 | ns |
| IL111 vs IL4457 | -1 | 0.917 | ns |
| IL111 vs IL4141 | -1.317 | 1.261 | ns |
| IL111 vs IL519 | -1.483 | 1.421 | ns |
| IL111 vs IL2156 | -2.233 | 2.139 | ns |
| IL111 vs IL521 | -2.817 | 2.698 | ns |
| IL111 vs IL3438 | -3.9 | 3.735 | * |
| IL111 vs IL2689 | -3.9 | 3.576 | ns |
| IL3912 vs IL4457 | -0.7667 | 0.7343 | ns |
| IL3912 vs IL4141 | -1.083 | 1.088 | ns |
| IL3912 vs IL519 | -1.25 | 1.256 | ns |
| IL3912 vs IL2156 | -2 | 2.009 | ns |
| IL3912 vs IL521 | -2.583 | 2.595 | ns |
| IL3912 vs IL3438 | -3.667 | 3.683 | ns |
| IL3912 vs IL2689 | -3.667 | 3.512 | ns |
| IL4457 vs IL4141 | -0.3167 | 0.3033 | ns |
| IL4457 vs IL519 | -0.4833 | 0.4629 | ns |
| IL4457 vs IL2156 | -1.233 | 1.181 | ns |
| IL4457 vs IL521 | -1.817 | 1.74 | ns |
| IL4457 vs IL3438 | -2.9 | 2.778 | ns |
| IL4457 vs IL2689 | -2.9 | 2.659 | ns |
| IL4141 vs IL519 | -0.1667 | 0.1674 | ns |
| IL4141 vs IL2156 | -0.9167 | 0.9208 | ns |
| IL4141 vs IL521 | -1.5 | 1.507 | ns |
| IL4141 vs IL3438 | -2.583 | 2.595 | ns |
| IL4141 vs IL2689 | -2.583 | 2.474 | ns |
| IL519 vs IL2156 | -0.75 | 0.7534 | ns |
| IL519 vs IL521 | -1.333 | 1.339 | ns |
| IL519 vs IL3438 | -2.417 | 2.428 | ns |
| IL519 vs IL2689 | -2.417 | 2.315 | ns |
| IL2156 vs IL521 | -0.5833 | 0.586 | ns |
| IL2156 vs IL3438 | -1.667 | 1.674 | ns |
| IL2156 vs IL2689 | -1.667 | 1.596 | ns |
| IL521 vs IL3438 | -1.083 | 1.088 | ns |
| IL521 vs IL2689 | -1.083 | 1.038 | ns |
| IL3438 vs IL2689 | 0 | 0 | ns |
|  |  |  |  |
| **CC lines** | **Change in body weight day 1** | | |
|  | **Mean Diff.** | **t** | **Significance** |
| IL519 vs IL521 | -4.135 | 3.81 | * |
| IL519 vs IL111 | -1.688 | 1.483 | ns |
| IL519 vs IL611 | -3.317 | 2.914 | ns |
| IL519 vs IL711 | -3.374 | 2.781 | ns |
| IL519 vs IL2126 | -2.654 | 2.446 | ns |
| IL519 vs IL2156 | -3.051 | 2.811 | ns |
| IL519 vs IL2689 | -3.758 | 3.301 | ns |
| IL519 vs IL3438 | -3.278 | 3.02 | ns |
| IL519 vs IL3912 | -3.278 | 3.02 | ns |
| IL519 vs IL4052 | -3.78 | 3.483 | ns |
| IL519 vs IL4141 | -1.263 | 1.164 | ns |
| IL519 vs IL4457 | -4.142 | 3.639 | ns |
| IL519 vs IL72 | -5.321 | 4.003 | * |
| IL519 vs IL188 | 1.146 | 0.8619 | ns |
| IL519 vs IL1912 | -2.183 | 1.799 | ns |
| IL519 vs IL1061 | -2.782 | 2.293 | ns |
| IL521 vs IL111 | 2.447 | 2.15 | ns |
| IL521 vs IL611 | 0.8184 | 0.7189 | ns |
| IL521 vs IL711 | 0.7609 | 0.6271 | ns |
| IL521 vs IL2126 | 1.481 | 1.364 | ns |
| IL521 vs IL2156 | 1.084 | 0.9989 | ns |
| IL521 vs IL2689 | 0.377 | 0.3312 | ns |
| IL521 vs IL3438 | 0.8573 | 0.7899 | ns |
| IL521 vs IL3912 | 0.8573 | 0.7899 | ns |
| IL521 vs IL4052 | 0.3551 | 0.3272 | ns |
| IL521 vs IL4141 | 2.872 | 2.646 | ns |
| IL521 vs IL4457 | -0.006963 | 0.006117 | ns |
| IL521 vs IL72 | -1.186 | 0.8925 | ns |
| IL521 vs IL188 | 5.281 | 3.973 | * |
| IL521 vs IL1912 | 1.952 | 1.609 | ns |
| IL521 vs IL1061 | 1.353 | 1.115 | ns |
| IL111 vs IL611 | -1.629 | 1.37 | ns |
| IL111 vs IL711 | -1.686 | 1.337 | ns |
| IL111 vs IL2126 | -0.9666 | 0.8491 | ns |
| IL111 vs IL2156 | -1.363 | 1.198 | ns |
| IL111 vs IL2689 | -2.07 | 1.741 | ns |
| IL111 vs IL3438 | -1.59 | 1.397 | ns |
| IL111 vs IL3912 | -1.59 | 1.397 | ns |
| IL111 vs IL4052 | -2.092 | 1.838 | ns |
| IL111 vs IL4141 | 0.4247 | 0.3731 | ns |
| IL111 vs IL4457 | -2.454 | 2.064 | ns |
| IL111 vs IL72 | -3.634 | 2.647 | ns |
| IL111 vs IL188 | 2.834 | 2.064 | ns |
| IL111 vs IL1912 | -0.4948 | 0.3924 | ns |
| IL111 vs IL1061 | -1.094 | 0.8677 | ns |
| IL611 vs IL711 | -0.05744 | 0.04555 | ns |
| IL611 vs IL2126 | 0.6623 | 0.5818 | ns |
| IL611 vs IL2156 | 0.2657 | 0.2334 | ns |
| IL611 vs IL2689 | -0.4414 | 0.3713 | ns |
| IL611 vs IL3438 | 0.03895 | 0.03422 | ns |
| IL611 vs IL3912 | 0.03895 | 0.03422 | ns |
| IL611 vs IL4052 | -0.4632 | 0.407 | ns |
| IL611 vs IL4141 | 2.054 | 1.804 | ns |
| IL611 vs IL4457 | -0.8253 | 0.6942 | ns |
| IL611 vs IL72 | -2.005 | 1.46 | ns |
| IL611 vs IL188 | 4.462 | 3.25 | ns |
| IL611 vs IL1912 | 1.134 | 0.8993 | ns |
| IL611 vs IL1061 | 0.5346 | 0.424 | ns |
| IL711 vs IL2126 | 0.7197 | 0.5931 | ns |
| IL711 vs IL2156 | 0.3232 | 0.2663 | ns |
| IL711 vs IL2689 | -0.384 | 0.3045 | ns |
| IL711 vs IL3438 | 0.09639 | 0.07944 | ns |
| IL711 vs IL3912 | 0.09639 | 0.07944 | ns |
| IL711 vs IL4052 | -0.4058 | 0.3344 | ns |
| IL711 vs IL4141 | 2.111 | 1.74 | ns |
| IL711 vs IL4457 | -0.7679 | 0.6089 | ns |
| IL711 vs IL72 | -1.947 | 1.356 | ns |
| IL711 vs IL188 | 4.52 | 3.148 | ns |
| IL711 vs IL1912 | 1.191 | 0.8963 | ns |
| IL711 vs IL1061 | 0.5921 | 0.4454 | ns |
| IL2126 vs IL2156 | -0.3966 | 0.3654 | ns |
| IL2126 vs IL2689 | -1.104 | 0.9696 | ns |
| IL2126 vs IL3438 | -0.6233 | 0.5743 | ns |
| IL2126 vs IL3912 | -0.6233 | 0.5743 | ns |
| IL2126 vs IL4052 | -1.126 | 1.037 | ns |
| IL2126 vs IL4141 | 1.391 | 1.282 | ns |
| IL2126 vs IL4457 | -1.488 | 1.307 | ns |
| IL2126 vs IL72 | -2.667 | 2.006 | ns |
| IL2126 vs IL188 | 3.8 | 2.859 | ns |
| IL2126 vs IL1912 | 0.4717 | 0.3888 | ns |
| IL2126 vs IL1061 | -0.1277 | 0.1052 | ns |
| IL2156 vs IL2689 | -0.7071 | 0.6212 | ns |
| IL2156 vs IL3438 | -0.2268 | 0.2089 | ns |
| IL2156 vs IL3912 | -0.2268 | 0.2089 | ns |
| IL2156 vs IL4052 | -0.729 | 0.6716 | ns |
| IL2156 vs IL4141 | 1.788 | 1.647 | ns |
| IL2156 vs IL4457 | -1.091 | 0.9585 | ns |
| IL2156 vs IL72 | -2.27 | 1.708 | ns |
| IL2156 vs IL188 | 4.197 | 3.157 | ns |
| IL2156 vs IL1912 | 0.8683 | 0.7156 | ns |
| IL2156 vs IL1061 | 0.2689 | 0.2216 | ns |
| IL2689 vs IL3438 | 0.4804 | 0.422 | ns |
| IL2689 vs IL3912 | 0.4804 | 0.422 | ns |
| IL2689 vs IL4052 | -0.02182 | 0.01917 | ns |
| IL2689 vs IL4141 | 2.495 | 2.192 | ns |
| IL2689 vs IL4457 | -0.3839 | 0.3229 | ns |
| IL2689 vs IL72 | -1.563 | 1.139 | ns |
| IL2689 vs IL188 | 4.904 | 3.572 | ns |
| IL2689 vs IL1912 | 1.575 | 1.249 | ns |
| IL2689 vs IL1061 | 0.9761 | 0.774 | ns |
| IL3438 vs IL3912 | 0 | 0 | ns |
| IL3438 vs IL4052 | -0.5022 | 0.4627 | ns |
| IL3438 vs IL4141 | 2.015 | 1.856 | ns |
| IL3438 vs IL4457 | -0.8643 | 0.7593 | ns |
| IL3438 vs IL72 | -2.044 | 1.537 | ns |
| IL3438 vs IL188 | 4.423 | 3.328 | ns |
| IL3438 vs IL1912 | 1.095 | 0.9025 | ns |
| IL3438 vs IL1061 | 0.4957 | 0.4085 | ns |
| IL3912 vs IL4052 | -0.5022 | 0.4627 | ns |
| IL3912 vs IL4141 | 2.015 | 1.856 | ns |
| IL3912 vs IL4457 | -0.8643 | 0.7593 | ns |
| IL3912 vs IL72 | -2.044 | 1.537 | ns |
| IL3912 vs IL188 | 4.423 | 3.328 | ns |
| IL3912 vs IL1912 | 1.095 | 0.9025 | ns |
| IL3912 vs IL1061 | 0.4957 | 0.4085 | ns |
| IL4052 vs IL4141 | 2.517 | 2.319 | ns |
| IL4052 vs IL4457 | -0.3621 | 0.3181 | ns |
| IL4052 vs IL72 | -1.542 | 1.16 | ns |
| IL4052 vs IL188 | 4.926 | 3.706 | ns |
| IL4052 vs IL1912 | 1.597 | 1.316 | ns |
| IL4052 vs IL1061 | 0.9979 | 0.8224 | ns |
| IL4141 vs IL4457 | -2.879 | 2.529 | ns |
| IL4141 vs IL72 | -4.058 | 3.053 | ns |
| IL4141 vs IL188 | 2.409 | 1.812 | ns |
| IL4141 vs IL1912 | -0.9196 | 0.7578 | ns |
| IL4141 vs IL1061 | -1.519 | 1.252 | ns |
| IL4457 vs IL72 | -1.179 | 0.8591 | ns |
| IL4457 vs IL188 | 5.288 | 3.852 | * |
| IL4457 vs IL1912 | 1.959 | 1.554 | ns |
| IL4457 vs IL1061 | 1.36 | 1.078 | ns |
| IL72 vs IL188 | 6.467 | 4.213 | * |
| IL72 vs IL1912 | 3.139 | 2.186 | ns |
| IL72 vs IL1061 | 2.539 | 1.769 | ns |
| IL188 vs IL1912 | -3.328 | 2.318 | ns |
| IL188 vs IL1061 | -3.928 | 2.736 | ns |
| IL1912 vs IL1061 | -0.5994 | 0.4509 | ns |
